# Understanding the element segregation and phase separation in the Ce-substituted Nd-(Fe,Co)-B based alloys

**DOI:** 10.1038/s41598-018-25230-0

**Published:** 2018-05-01

**Authors:** L. Z. Zhao, J. S. Zhang, G. Ahmed, X. F. Liao, Z. W. Liu, J. M. Greneche

**Affiliations:** 1 School of Materials Science and Engineering, South China University of Technology, Guangzhou, 510640 China; 2 Innovative Center for Advanced Materials (ICAM), Hangzhou Dianzi University, Hangzhou, 310012 China; 3 Institut des Molécules et Matériaux du Mans CNRS UMR-6283, Le Mans Université, Avenue Messiaen, Le Mans, F-72085 France

## Abstract

Ce substituted Nd_2_Fe_14_B (2:14:1)-type permanent magnets have shown increasing potential in the applications due to their high properties/cost ratio. However, the element segregation and phase separation in the Ce substituted magnets have not been fully understood yet. In this work, (Nd_1−x_Ce_x_)_25_Fe_40_Co_20_Al_4_B_11_ alloys with high coercivities were prepared by copper mold casting. Based on detailed microstructure and composition analysis, the segregation of rare earth (RE) elements was observed in the as-cast alloys. Nd element prefers to enter into the 2:14:1 phase and the Ce element enter into the 1:2 phase. The existence of the 1:2 phase can promote the element segregation. The alloy shows an abnormal increase of coercivity from 641 kA/m for x = 0.2 to 863 kA/m for x = 0.3. This increase could be attributed to the phase separation of the 2:14:1 phase, which has been confirmed by the microstructural characterization. The present data provides useful information for exploring Ce-containing Nd-Fe-B magnets.

## Introduction

Nd–Fe–B based alloys have been widely used as the most powerful permanent magnets for the last 30 years. The replacement of Nd by Ce element has been suggested as an alternative to reduce the global cost of the permanent magnets^1,2^. Even though the intrinsic properties of the Ce_2_Fe_14_B phase (*J*_s_ = 1.17 T, *H*_a_ = 30 kOe and *T*_c_ = 442 K) are relatively lower compared to those of Nd_2_Fe_14_B (2:14:1) phases^3^. Some experimental studies suggested that up to 30 at.% substitution of Ce for Nd in Nd-Fe-B alloys did not destroy the magnetic properties, but further increase of Ce content would reduce the hard magnetic properties significantly^4,5^. Particularly, several topics deserve more attention in the case of Ce-substituted Nd-Fe-B magnets. First, when the Nd is partially substituted by other rare earth (RE), the RE elements show their preference to enter into certain phase due to substitution energy variation. It was reported that the Y, Tb and Dy elements prefer to enter the 2:14:1 phase but avoid the Nd-rich phase, while the La and Ce ones exhibit the opposite preference^6–8^. Thus, it is crucial to explore the influence of the Ce elements on magnetic properties and to control their distribution in the Ce-substituted magnets. Second, the previous studies have shown that REFe_2_ phase is easy to precipitate in the Ce-Fe-B and Ce-containing Nd-Fe-B alloys^9–11^. The CeFe_2_ phase behaves as a soft magnetic phase below 230 (±2) K^12^ and its presence is harmful to the hard magnetic properties. On the other hand, REFe_2_ phase also brings some benefits for the sintered magnets. Zhang *et al*.^10^ reported that the REFe_2_ phase could improve the wettability of 2:14:1 phase when the magnets are sintered at temperature above the melting point of REFe_2_ phase. Our previous work also suggested that Ce atom prefer to enter into the 1:2 phase, which leads the element segregation and increases the Nd content in the (NdCe)_2_Fe_14_B phase^8^. Third, Capehart *et al*. reported a mixed-valent state with the coexistence of Ce^3+^ and Ce^4+^ atoms in the Ce_2_Fe_14_B phase based on the evidence provided by Ce L_3_-edge x-ray absorption near-edge structure measurement^13^. Their steric variation combined with different electron density give rise to the valence state dependent magnetic and metallurgical behaviors of Ce-Nd-Fe-B alloys^14,15^. The last but not the least, an abnormal increase of coercivity has been observed in the Ce-Nd-Fe-B alloys with around 25% Ce-substitution, and several researchers have attributed it to the phase separation^2,16–18^. However, to the best of our knowledge, no microstructural evidence of the phase separation has been reported yet. While, the recent study on the Ce-substituted single crystal indicated a disagreement^19^, which causes more doubts on the phase separation.

In this work, aiming at understanding the element segregation and phase separation in the Ce-substituted alloys, the microstructure and the magnetic properties of the directly cast Ce-substituted Nd-Fe-B based alloys have been investigated. The segregation of RE elements, the roles of REFe_2_ phase, the mixed valence state of Ce, and the phase separation of 2:14:1 phase have been carefully investigated and discussed. In particular, the phase separation is confirmed directly by novel microstructural analysis. Here, directly cast (Nd_1−x_Ce_x_)_25_Fe_40_Co_20_Al_4_B_11_ alloys are chosen because their grain sizes are in micrometer scale, which can be easily characterized by scanning electron microscopy and energy dispersive spectrometry. In addition, those cast alloys have high coercivities, which is beneficial for revealing the relationships between the magnetic properties and microstructures^20,21^. The present work may help to further understand the structure and magnetic properties of Ce-containing Nd-Fe-B alloys.

## Results

### Phase constitution and microstructure

**Figure 1:**
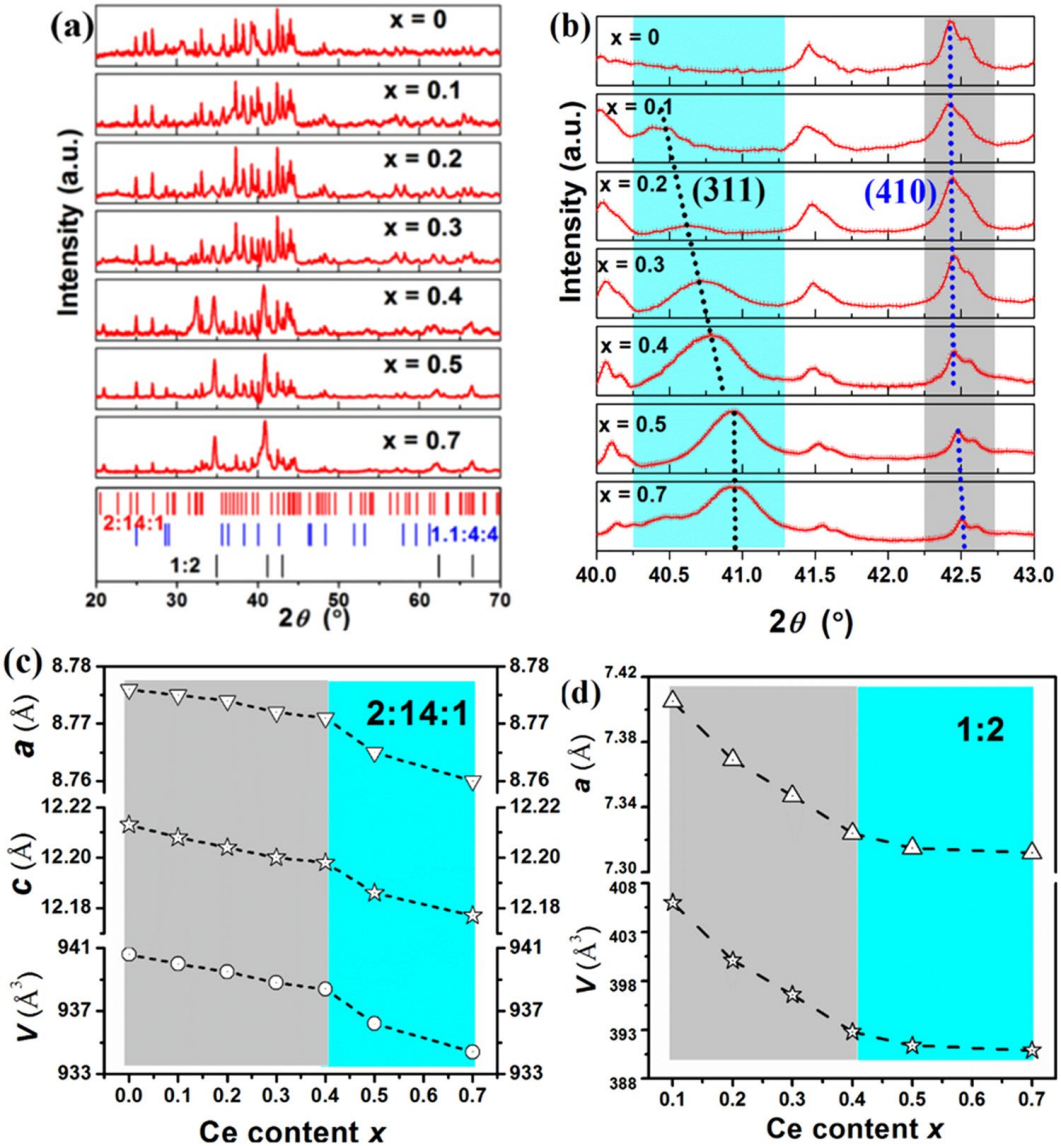
XRD patterns for the as-cast (Nd_1−x_Ce_x_)_25_Fe_40_Co_20_Al_4_B_11_ alloys (**a**), the enlarged patterns in the range of 40–43° (**b**), and the calculated cell parameters of 2:14:1 phase (**c**) and 1:2 phase (**d**) changed with Ce content *x*.

The XRD results of selected (Nd_1−x_Ce_x_)_25_Fe_40_Co_20_Al_4_B_11_ samples with various Ce substitutions for Nd are showed in Fig. 1a. As discussed in our previous work^21^, the x = 0 sample is composed of Nd_2_(FeCoAl)_14_B phase (2:14:1 phase) with a space group of P_42_/mnm and Nd_1+ε_(FeCo)_4_B_4_ phase (1:4:4 phase) with a space group of P4_2_/n. With substitution of Ce for Nd, the 1:4:4 phase is replaced by the (NdCe)(FeCo)_2_ phase (1:2 phase) with a space group of Fd$\overline{{\rm{3}}}$m. With increasing Ce content, the relative intensity of the 1:2 phase increases, indicating that the content of the 1:2 phase in the sample increases. Figure 1b shows the XRD patterns in the 2θ-range of 40–43°. The marked dotted lines in Fig. 1b show that the peaks shift to higher angles with the increasing Ce content. Refined values of cell parameters of 2:14:1 and 1:2 phases are plotted with *x* in Fig. 1c and d, respectively. The lattice parameters of the 2:14:1 phase and the 1:2 phase decrease monotonically, but not linearly, with the increasing Ce content. For the 2:14:1 phase, the values of *a*, *c*, and *V* decrease slowly from x = 0 to x = 0.4 at first, and then drop more quickly for x > 0.4. For the 1:2 phase, an opposite change tendency is observed, and the values of *a* and *V* decrease quickly from x = 0 to x = 0.4 but decrease slowly between x = 0.5 and 0.7. From these data, it could be expected that the magnetic properties would have changed accordingly since the strength of the magnetic coupling and anisotropy are correlated to the distance between magnetic atoms, which is dependent on the cell parameters.

**Figure 2:**
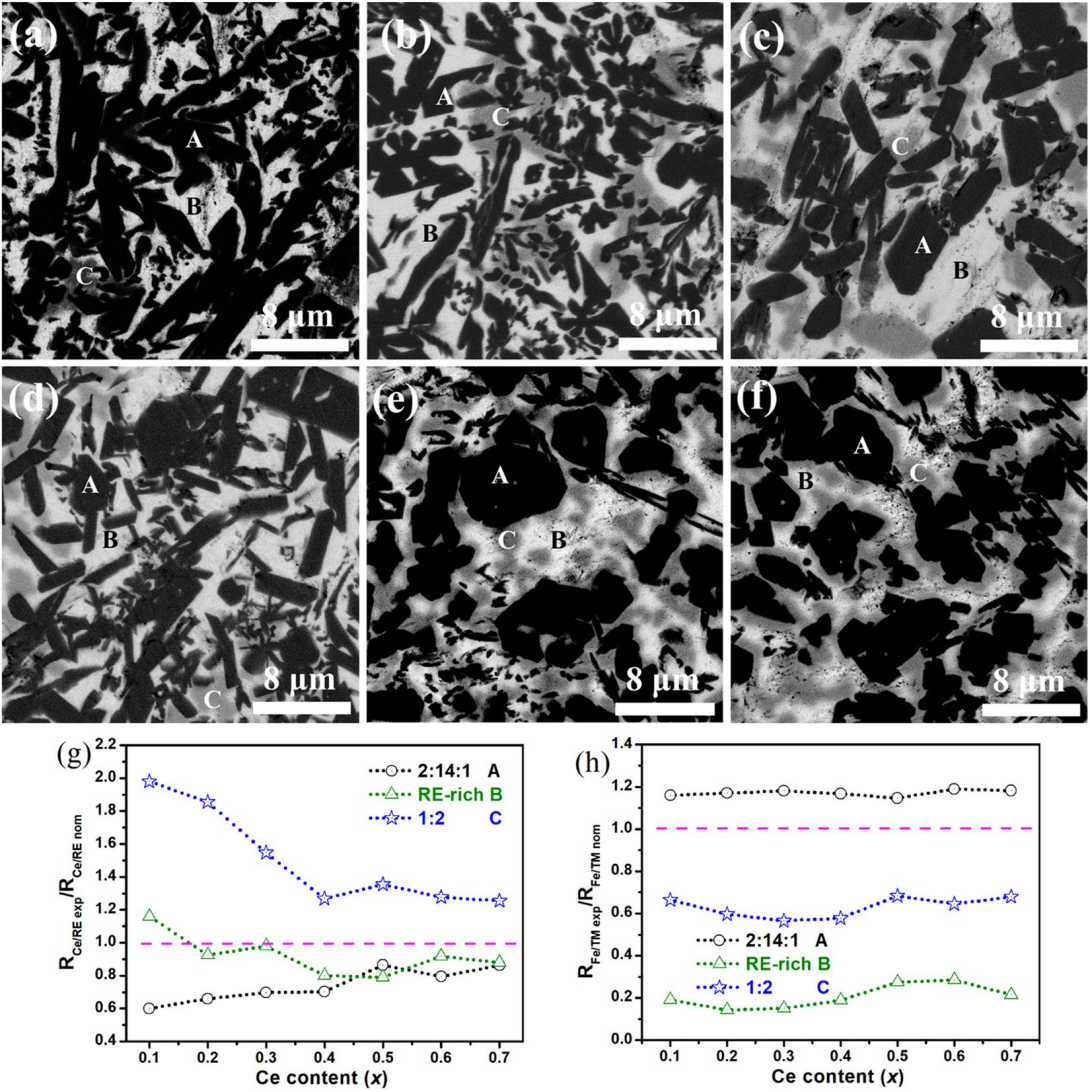
Back-scattered SEM images for the as-cast (Nd_1−x_Ce_x_)_25_Fe_40_Co_20_Al_4_B_11_ alloys with (**a**) x = 0, (**b**) x = 0.2, (**c**) x = 0.3, (**d**) x = 0.4, (**e**) x = 0.5, and (**f**) x = 0.7, and the dependences of R_Ce/RE exp_/R_Ce/RE nom_ (**g**) and R_Fe/TM exp_/R_Fe/TM nom_ (**h**) ratios on Ce content *x* for different phases.

The back-scattered SEM images for the selected samples are shown in Fig. 2a–f. Three different contrasts, marked as area A, B, and C in all figures, can be observed. For the x = 0 sample (Fig. 2a) without Ce substitution, as discussed in our previous work^20^, area A is represented for 2:14:1 phase, B is for Nd-rich phase while C (dark gray) for 1:4:4 phase. For the Ce-substituted alloys, according to the EDS results shown in the supplementary materials as Table S1, the ratio of rare earth Nd + Ce content over transition metal Fe + Co content (RE/TM_atom_) is near to 1:7 for point A, 2:1 for point B, and 1:2 for point C, which should correspond to 2:14:1 phase, RE-rich phase, and 1:2 phase, respectively. It is found that when the alloy was substituted by Ce, the 1:4:4 phase disappeared while the 1:2 phase appeared. The area fraction of the 1:2 phase increases with the increasing Ce content, in agreement with the XRD results. The distribution of the 1:2 phase also changes with Ce content. For the x = 0.1–0.4 alloys, as displayed in Fig. 2b–d, the 1:2 phases are randomly distributed. In the case of x = 0.5–0.7, as illustrated in Fig. 2e and f, the 1:2 phase prefers to stay around the 2:14:1 phase, giving rise to a ‘core-shell’ like structure.

Based on the EDS results, the experimental Ce/RE and Fe/TM ratios in the 2:14:1, RE-rich, and 1:2 phases are compared with nominal Ce/RE and Fe/TM ratios, the R_Ce/RE exp_/R_Ce/RE nom_ and R_Fe/TM exp_/R_Fe/TM nom_ changing with Ce content x were plotted in Fig. 2g and h, respectively. In Fig. 2g, the line *y* = 1 is the standard line. For the 2:14:1 phase, all the values of R_Ce/RE exp_/R_Ce/RE nom_ are lower than 1, which means that the Nd atoms prefer to enter into the 2:14:1 phase. By contrast, all the values of R_Ce/RE exp_/R_Ce/RE nom_ in the 1:2 phase are higher than 1, indicating a Ce segregation. For the RE-rich phase, in the x = 0.1–0.3 alloys, the values of R_Ce/RE exp_/R_Ce/RE nom_ are close to 1, but it starts to be lower for x > 0.3, suggesting the Nd segregation. The results thus indicate a strong RE element segregation in the alloys. Ce atoms prefer to enter into 1:2 phase, but expel from the 2:14:1 phase, and the distribution in RE-rich phase is dependent on the Ce content. Figure 2h presents the R_Fe/TM exp_/R_Fe/TM nom_ in the various phases, and the y = 1 is the standard line. One could find that the segregation of TM also occurs. Fe atoms prefer to enter into 2:14:1 phase, but to expel from both RE-rich and 1:2 phases. On the other hand, the Fe/TM ratios in those three phases are independent of the Ce content.

**Figure 3:**
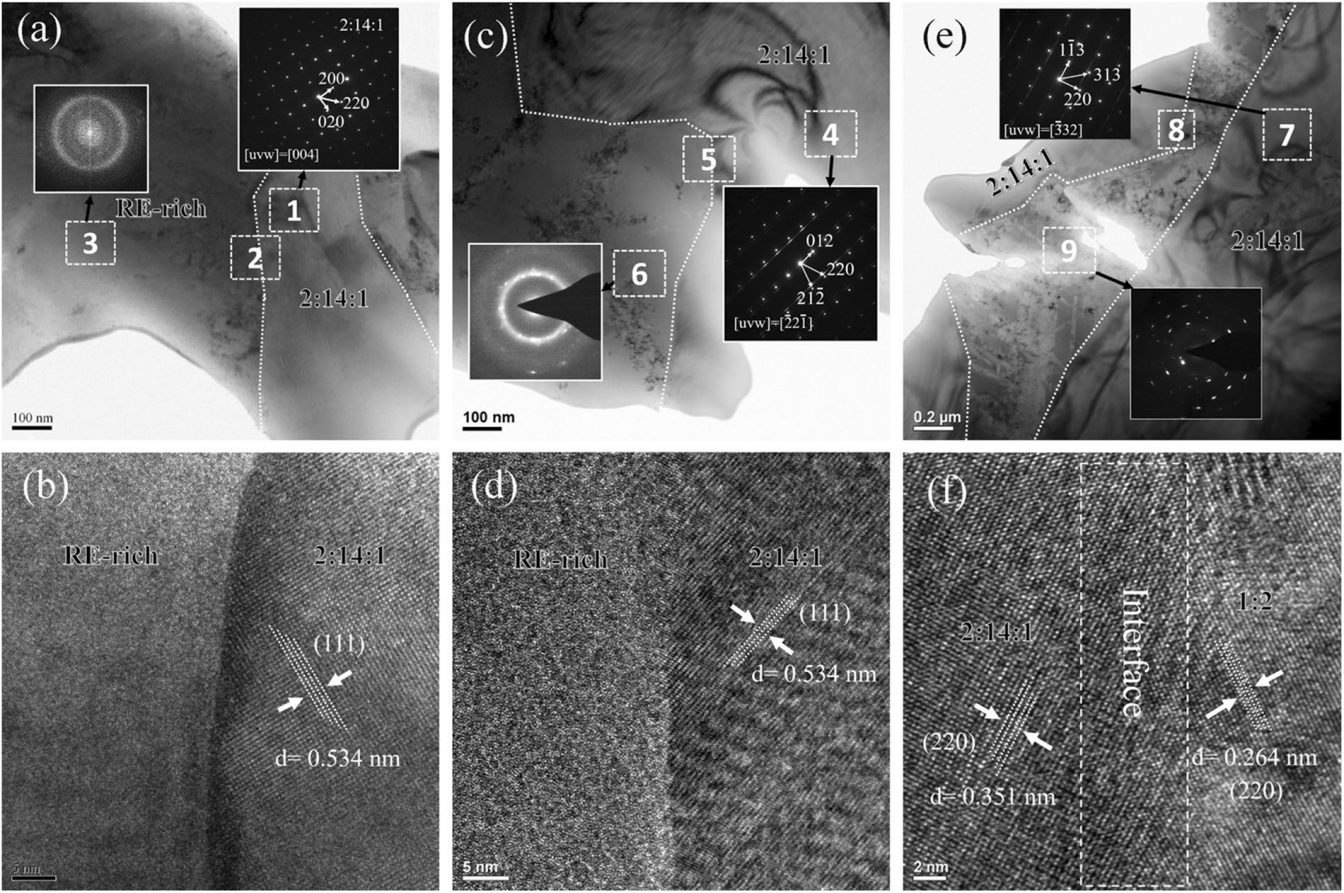
TEM images for the selected as-cast (Nd_1−x_Ce_x_)_25_Fe_40_Co_20_Al_4_B_11_ alloys (**a**), (**b**) x = 0; (**c**,**d**) x = 0.3; (**e**,**f**) x = 0.7.

The selected samples are characterized by TEM. Figure 3a illustrates a micrometer sized grain (labelled 1) and intergranular phases (labelled 3) for the x = 0 sample. The select area diffraction patterns (SADP) corresponding to areas 1 and 3 confirm the microstructure of 2:14:1 grains surrounded by amorphous RE-rich phase. Figure 3b shows the interface (labelled 2) between the 2:14:1 grain and the intergranular phase. The smooth interfacial structure is beneficial for the coercivity^22^. Similarly, the TEM image obtained from the x = 0.3 sample indicates that the micrometer sized grain (labelled 4) is surrounded by intergranular phase (labelled 6) (see Fig. 3c). However, according to the SADP image for the selected area 6, the intergranular phase could be indexed as nanocrystalline grains embedded in the amorphous matrix. Moreover, in the SADP image for selected area 4, except the main pattern of 2:14:1 phase with a $[\bar{2}2\bar{1}]$ crystal belt, a satellite pattern or, more concisely, a steak-line pattern, is also observed. Such a feature should be related to the phase separation^2^, as discussed later. The interfacial area labelled 5 in the HRTEM graph (Fig. 3d) shows the smooth grain boundary with the amorphous RE-rich phase, similar to that in the x = 0 sample. Figure 3e clearly shows that the structure of the intergranular phase for the x = 0.7 sample is more complicated than those of the x = 0 and x = 0.3 samples. The SADP image of area 9 suggest that the intergranular phase is mainly composed of nanocrystals and the 2:14:1 grains are surrounded by crystalline grains, consistently with the SEM results. Figure 3f is the HRTEM micrograph for the interfacial area 8, the right part of the graph shows an interplanar distance of 0.264 nm, corresponding to the [220] plane of 1:2 phase. It thus confirms that the surrounded phases are 1:2 phases, as discussed in Fig. 2, which form a ‘core-shell’ like structure with the 2:14:1 grains.

### Magnetic properties and hyperfine structure

**Figure 4:**
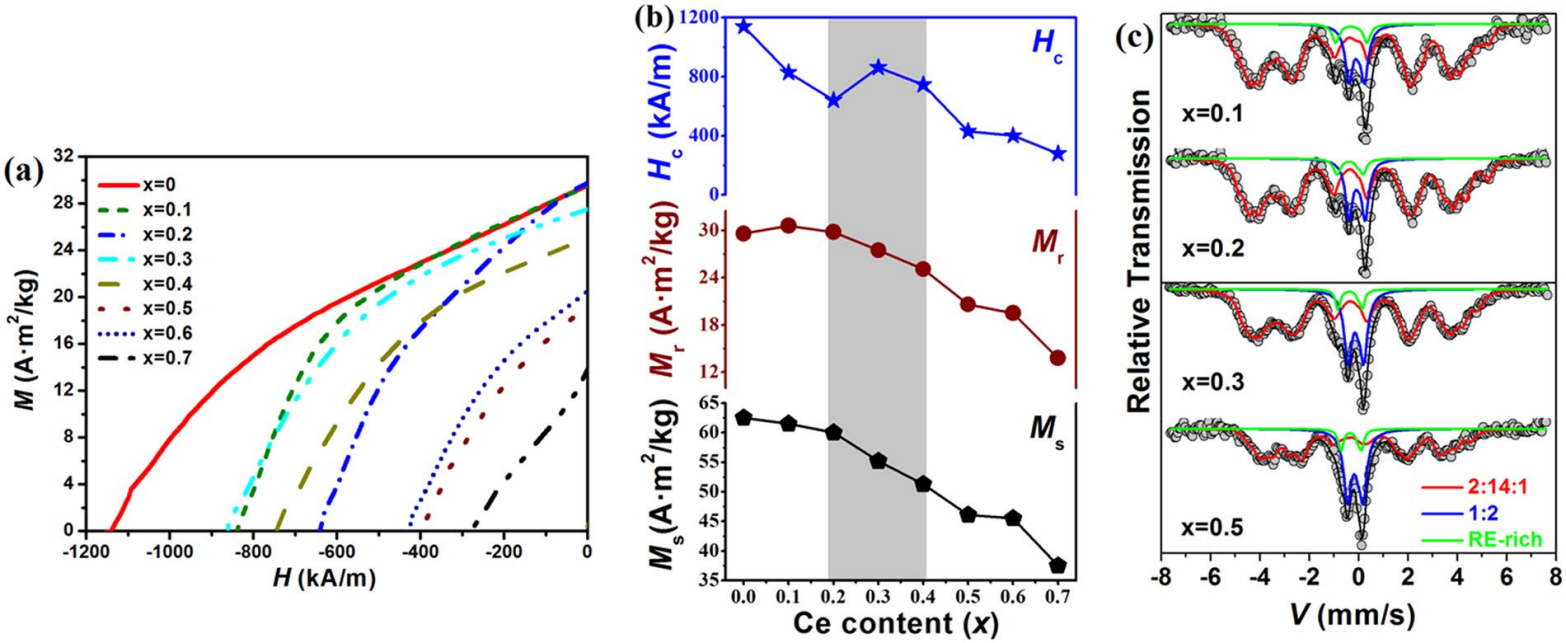
The demagnetization curves (**a**) and magnetic properties (**b**) for the as-cast (Nd_1−x_Ce_x_)_25_Fe_40_Co_20_Al_4_B_11_ alloys, and Mössbauer spectra at 300 K for the selected as-cast (Nd_1−x_Ce_x_)_25_Fe_40_Co_20_Al_4_B_11_ alloys (**c**).

The demagnetization curves for the alloys with various Ce contents are shown in Fig. 4a. All alloys exhibit a single phase-like hard magnetic behavior, and the demagnetization curve shrinks rapidly after 40% of Nd is substituted. As shown in Fig. 4b, for the low Ce contents (x < 0.2), both saturation magnetization *M*_s_ and remanence *M*_r_ remain almost constant, but drop monotonically for the increasing Ce content (x > 0.2). Since all samples are magnetically isotropic, the *M*_r_/*M*_s_ values all keep close to 1/2. It has been demonstrated that the volume fraction of 1:2 phase and the Ce/RE% of 2:14:1 phase increase with the increasing Ce content, which should be the reasons for the degradation of both *M*_r_ and *M*_s_. The (*BH*)_max_, not shown here, also shows the same trend as the change of *M*_s_. Interestingly, intrinsic coercivity *H*_ci_ increases unexpectedly at x = 0.3, similar to those previously observed by various researchers^2,16^. It increases from 641 kA/m for x = 0.2 to 863 kA/m for x = 0.3, which is still higher than the 829 kA/m of the x = 0.1 sample. After x > 0.4, the rapid decrease of the coercivity could be attributed to the “core-shell” like structure discussed above^22^.

^57^Fe Mössbauer spectrometry was employed to investigate the hyperfine structures of the different alloys and to discriminate their Fe-based phases. The spectra registered at 300 K are shown in Fig. 4c and the refined values of hyperfine parameters are listed in Table 1. The hyperfine structures for all samples are quite complicated and have to be described by at least three main components, including one consisting of six sextets to describe the 2:14:1 phase, and two quadrupolar doublets assigned to the paramagnetic RE-rich phase and the 1:2 phase. The assignment of the six magnetic components is based upon the Wigner-Seitz analysis of the RE_2_Fe_l4_B structure^23^, which is even more complicated in the presence of Co^24^. The refined values of hyperfine parameters of the CeFe_2_ phase are consistent with those previously reported^11,25^ and the remaining paramagnetic component was assigned to the RE-rich phase. From Table 1, the content of the 1:2 phase increases with the increasing Ce content at the expense of the 2:14:1 phase, while that of the RE-rich phase remains almost independent with Ce content. The 2:14:1 phase is the only magnetic phase in the alloys at 300 K and its magnetic moments are from 14 transition metal (TM) atoms Fe and Co, and 2 rare earth (RE) atoms Nd and Ce. It was reported that the magnetic moment of the Ce atom in the 2:14:1 structure was extremely low, ranging 0.1 ~ 0.5 μ_B_^11,15^. According to the mean hyperfine field of the Fe atom (Table 1), and by using the rule of 15 T/μ_B_^23^, the mean magnetic moment of Fe atoms can be estimated at 1.70 μ_B_, 1.68 μ_B_, 1.67 μ_B_, and 1.55 μ_B_ (±0.01 μ_B_) for the x = 0.1, 0.2, 0.3, and 0.5 alloys, respectively^23^. It is clear that, with Ce substitution for Nd, the magnetic moment exhibits a minor decrease from x = 0.1 to 0.3, then a rapid decrease afterward. Thus, due to the decrease of the Fe moment with Ce substitution and the substitution of magnetic Nd (3.2 μ_B_) by near non-magnetic Ce^3^, the magnetic moment of the 2:14:1 phase decreases with the increasing Ce content, especially for x > 0.3.

**Table 1:** Refined values of the hyperfine parameters at 300 K for the selected as-cast (Nd_1−x_Ce_x_)_25_Fe_40_Co_20_Al_4_B_11_ alloys.

Sample	Phase	*IS* (mm/s) (±0.03)	2ε/*QS* (mm/s) (±0.03)	*B*_hf_(T) (±0.1)	% (±2)
x = 0.1	2:14:1	〈−0.05〉	〈0.24〉	〈25.6〉	81
	1:2	0.07	0.59	0	15
	RE-rich	−0.22	0.99	0	4
x = 0.2	2:14:1	〈−0.06〉	〈0.20〉	〈25.2〉	79
	1:2	0.06	0.57	0	17
	RE-rich	−0.25	1.00	0	4
x = 0.3	2:14:1	〈−0.06〉	〈0.2〉	〈25.0〉	77
	1:2	0.02	0.57	0	19
	RE-rich	−0.21	0.92	0	4
x = 0.5	2:14:1	〈−0.01〉	〈0.22〉	〈23.3〉	65
	1:2	−0.01	0.55	0	29
	RE-rich	−0.18	0.76	0	6

## Discussions

To take the advantage of the RE elements doping in the Nd-Fe-B alloys, it is important to understand their partitioning in the main phase and the intergranular phase. Recently, Li *et al*.^26^ reported that Nd atoms tend to diffuse into the main phase during the Ce substitution in the strip cast (Nd_1−x_Ce_x_)_30_Fe_69_B (wt%) alloys, in agreement with our results in Fig. 2g.

The reason for Nd segregation in the 2:14:1 phase can be contributed to the substitution energy of RE (*E*_sub_) in 2:14:1 phase, which can be described as equation 1:

1 $${E}_{sub}={E}_{{(N{d}_{1-x}R{E}_{x})}_{2}F{e}_{14}B}-{E}_{N{d}_{2}F{e}_{14}B}-2x{E}_{RE}+2x{E}_{ND}$$

where *E*_(Nd1−xREx)2Fe14B_ represents the total energy at 0 K. The negative *E*_sub_ means that the substituted RE prefers to enter into 2:14:1 phase, while the positive *E*_sub_ indicates that the substitution will be expelled from the 2:14:1 phase. As reported by Liu *et al*.^6,7^, the *E*_sub_ for Y, Dy and Tb were negative, but for La it was positive (0.41 eV/atom). Since La and Ce elements are expected to have similar properties, and both La^3+^ and Ce^3+^ ionic radii are larger than that of Nd^3+^. Hence, the *E*_sub_ for Ce should be positive, and then it will be expelled from the 2:14:1 phase, as evidenced from the present experimental results.

However, this study gives different results from that of Li *et al*.^26^. Firstly, Li *et al*. reported that the Ce atoms preferred to enter into the RE-rich phase, which is *a priori* conflicting with the Nd segregation suggested in this work. We suggest here that the existence of REFe_2_ phase be responsible for Ce segregation. It was established that the large atom radius prevent to obtain stable NdFe_2_, LaFe_2_, PrFe_2_ and YbFe_2_ phases^27^. Cannon *et al*.^28^ also reported that the NdFe_2_, PrFe_2_ and YbFe_2_ phases could only be formed under high pressure and temperature (80 kbars, 1200 °C), except for CeFe_2_ phase due to its mixed valence state. The small radius of Ce^4+^ ion makes it easier to form the CeFe_2_ phase, which explains the Ce segregation into the 1:2 phase and thus also enhances the Nd segregation into the 2:14:1 phase. Secondly, in the strip cast (Nd_1−x_Ce_x_)_30_Fe_69_B(wt.%) alloys, Li *et al*. found that the Ce/RE ratio in 2:14:1 phase depended linearly on the Ce content, but in our case, as shown in Fig. 2g, the Ce/RE ratio increases more quickly for x > 0.4 in the 2:14:1 phase while the opposite tendency is observed in the 1:2 phase. To explain our result, the increase of the occupation results from the decrease of *E*_sub_, which can be achieved effectively by decreasing the cell volume^6^. In the Ce-based or Ce-substituted 2:14:1 phase with mixed valence state, i.e. the coexisting Ce^3+^ and Ce^4+^ states^13,17^, as Ce^4+^ ion is smaller than Ce^3+^ ion, *E*_sub_ will decrease when more Ce atoms are in the Ce^4+^ state, and consequently more Ce atoms will enter into 2:14:1 phase.

The changes of Ce/RE ratios in 2:14:1 and 1:2 phases lead to the variations of the cell parameters of both phases. As shown in Fig. 1c and d, the changes of the cell volume *V* can be correlated to the values of R_Ce/RE exp_/R_Ce/RE nom_ displayed in Fig. 2g and h. For the 2:14:1 phase, Ce/RE ratio in the *x* = 0–0.4 alloys increases more slowly than that in the x = 0.5–0.7 alloys, and as a result, the *V*_2:14:1_ of *x* = 0–0.4 alloys decreases more slowly. For x > 0.4, the quicker decrease of *V*_2:14:1_ is related to the quicker increase of Ce/RE ratio and also to that more Ce atoms in Ce^4+^ state. Similarly, the Fe magnetic moments of the 2:14:1 phase estimated from the Mössbauer results also show the same tendency. The weak decrease of magnetization at the early stage of Ce substitution indicates that the Ce-substituted magnets may be suitable for application.

**Figure 5:**
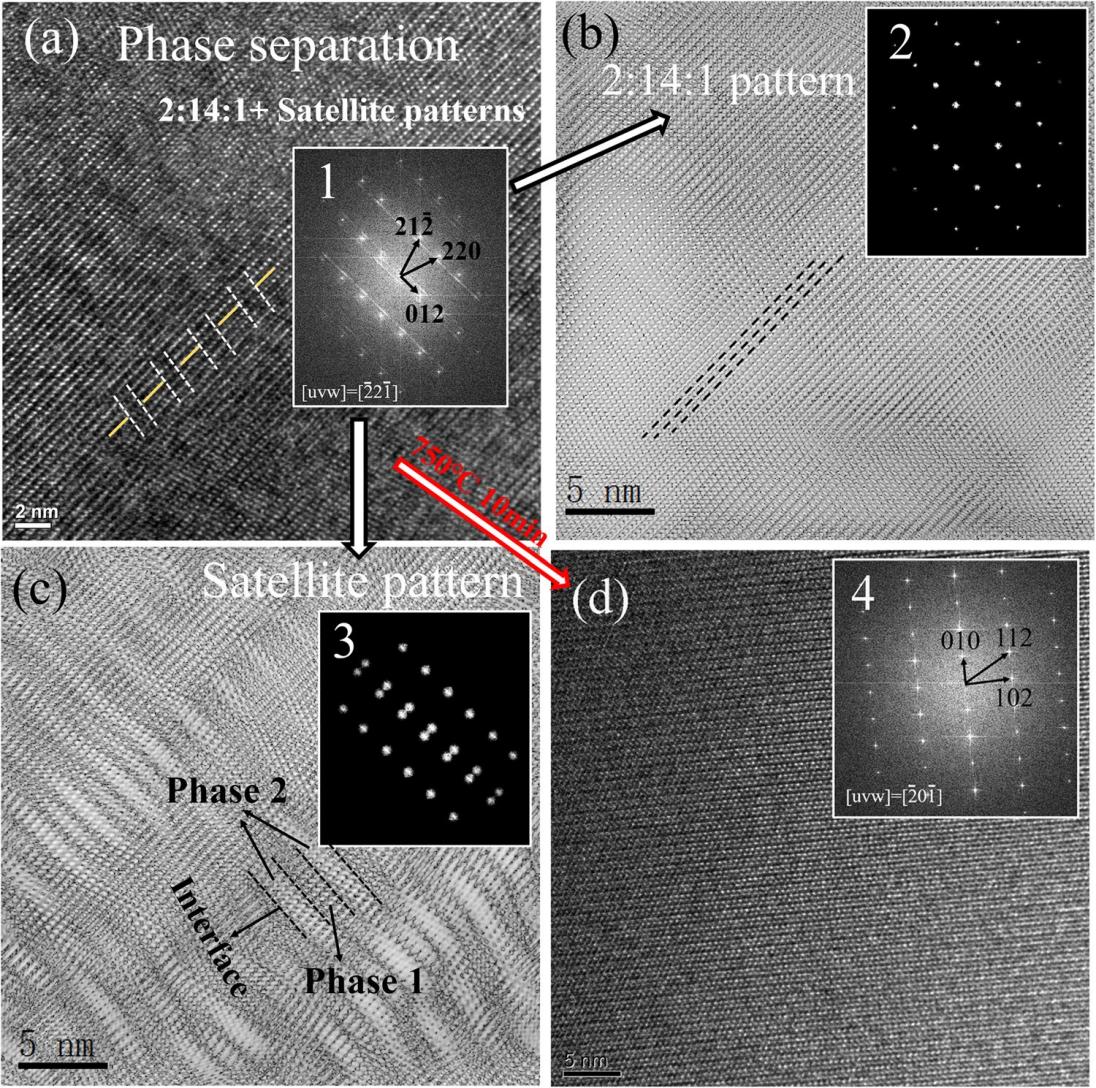
(**a**) HRTEM image and FFT image (inset 1) of the 2:14:1 phase in the as-cast *x* = 0.3 sample (the FFT image pattern can be divided into 2 patterns, i.e. a 2:14:1 main pattern (b inset 2) and a satellite pattern (b inset 3)); (**b**) the inverse FFT image for the main pattern in b inset 2; (**c**) the inverse FFT image for the satellite pattern in c inset 3; (**d**) the HRTEM image and FFT image (inset 4) of 2:14:1 phase in the as-cast x = 0.3 sample heat treated at 750 °C for 10 min.

The most interesting result in the present work is the microstructural evidence for the phase separation. As illustrated in Fig. 3c, the steak line diffraction pattern of 2:14:1 phase in the x = 0.3 sample indicates a two-phase structure in the alloy, which should be responsible for the abnormal increase of coercivity. The mechanism for phase separation in such alloys was investigated by Alam *et al*.^17^ and they found that when the Ce substitution exceeded 25%, the chemical inhomogeneity occurred in (NdCe)_2_Fe_14_B phase due to the significant difference in Nd_2_Fe_14_B and Ce_2_Fe_14_B lattice volumes. In our work, the phase separation is observed in the x = 0.3 sample, which has an actual Ce/RE ratio close to 21%. The HRTEM micrograph in Fig. 5a shows that the crystal planes of the 2:14:1 phase are not perfectly aligned for the x = 0.3 sample, different from the typical ones of Nd_2_Fe_14_B crystal^29,30^. The periodic differences are marked with dashed line in Fig. 5a. It can be assumed that each period corresponds to one phase, and the strain between the two neighboring phases creates the dislocations at the interfaces. The difference between those two phases may result from the Ce/RE ratio difference, one is higher than 21% (with lower cell volume) while the other one is lower than 21% (with higher cell volume), but both of them have the same 2:14:1 structure and crystal orientation. Wu *et al*.^31^ also reported the similar phenomenon observed at the interfaces of PbTe/PbS phases. Here we found this structure in all 2:14:1 phases of the x = 0.3 sample. The fast Fourier transformation (FFT) graph, shown as inset 1, presents a typical pattern for the 2:14:1 phase and a satellite pattern. Those two patterns can be separated by using ‘apply mask’ in the *DigitalMicrograph* software into the 2:14:1 pattern (inset 2) and satellite pattern (inset 3), and their inverse FFT graphs are illustrated in Fig. 5b and c, respectively. In Fig. 5c, the boundaries of the two-phase structure are clearly observed and it proves that the dislocations in the boundaries of those two phases are responsible for the steak-line diffraction pattern. To the best our knowledge, this nanoscale periodic two-phase structure with a width around 2 nm has never been reported before.

As predicted by Alam *et al*.^17^, this inhomogeneous structure would disappear at high temperature. Thus, the x = 0.3 sample was heat treated at 750 °C for 10 min. The HRTEM micrograph for the heat treated sample exhibits perfectly aligned crystal planes (Fig. 5d) and the FFT graph presents the typical single crystal diffraction pattern (inset 4). It means that the two-phase structure disappears after heat treatment. This study well explains why all the reported phase separation phenomena were only observed in the directly quenched alloys^2,16,18^, but not in the single crystal or sintered samples. Hence, our work will make this topic more conclusive^19,32,33^.

In summary, the (Nd_1−x_Ce_x_)_25_Fe_40_Co_20_Al_4_B_11_ (*x* = 0–0.7) alloys were prepared by copper mold casting and their composition-microstructure-magnetic properties relationships have been studied in this work in order to better understand the element segregation and phase separation in the Ce-substituted Nd-(Fe,Co)-B based alloys. In particular, the element segregation, mixed Ce valence states, roles of the REFe_2_ phase, phase separation, and high coercive field in the Ce-substituted alloys have been discussed in details. The cast Ce-substituted alloys are composed of 2:14:1 phase, 1:2 phase and RE-rich phase. The Ce/RE ratio is found lower than the nominal ratio in the 2:14:1 phase but higher in the 1:2 phase, indicating that Ce atoms prefer to enter 1:2 phase and tend to avoid the 2:14:1 phase due to the positive *E*_sub_. The weak increase of the Ce/RE ratio in the 2:14:1 at the early stage (*x* = 0–0.4) contributes to a slow decrease of the cell parameters and Fe magnetic moment for the 2:14:1 phase. But when *x* exceeds 0.4, the rapid decrease of the cell parameters could be attributed to more tetravalent Ce atoms. It is interesting to find the preliminary microstructural evidence for the phase separation in this work, which should be the reason for the abnormal increase of coercivity in the as-cast x = 0.3 sample.

## Methods

The (Nd_1−x_Ce_x_)_25_Fe_40_Co_20_Al_4_B_11_ (x = 0–0.7, atomic ratio) alloy ingots were prepared by arc-melting the mixture of pure metals Ce (99.9 wt.%), Nd (99.9 wt.%), Fe (99.9 wt.%), Co (99.9 wt.%), Al (99.9 wt.%), and B (99.9 wt.%) in an argon atmosphere. The ingots were re-melted 5 times to promote chemical composition homogeneity. Bulk rods with 2 mm in diameter were produced by injection casting the molten alloy into the copper mold without cooling water under the protection of Ar atmosphere. The selected x = 0.3 sample was annealed at 750 °C for 10 min in an argon atmosphere.

The magnetic properties were measured using physical property measurement system (PPMS-9, Quantum Design) equipped with a 9 T vibrating sample magnetometer (VSM). The microstructure was examined by transmission electron microscopy (TEM, Tecnai G2 F20 S-TWIN 200 kV) and scanning electron microscope backscattering electron mode (SEM, Nano430, FEI Co, BSE mode) equipped with an energy dispersive spectrometer (EDS). For the EDS results, at least 5 points were measured to get the mean values. The phase structure was determined by X-ray diffraction (XRD, Philips X-pert) with Cu-K_α_ radiation. The alloys were investigated by ^57^Fe Mössbauer spectrometry (MS), and the spectra were recorded at 300 K in a transmission geometry using ^57^Co/Rh *γ*-ray source mounted on an electromagnetic drive with a triangular velocity form. For both the XRD and MS measurement, the rod-shaped samples had to be crushed into fine powders to prevent from any preferential and thickness effects.

## Electronic supplementary material

Table S1
