# Supplementary material for: Understanding the element segregation and phase separation in the Ce-substituted Nd-(Fe,Co)-B based alloys
Source: Sci Rep. 2018 May 1;8:6826. doi: 10.1038/s41598-018-25230-0 (PMC5931561; doi:10.1038/s41598-018-25230-0)
Supplement: Supplementary file 1 — Table S1 [file 41598_2018_25230_MOESM1_ESM.pdf]

Understanding the element segregation and phase separation in the Ce-substituted Nd-(Fe,Co)-B based alloys

L.Z Zhao[1,2,*], J.S. Zhang[1], G. Ahmed[1], X.F. Liao[1], Z.W. Liu[1,*], J.M. Greneche[3]
1. School of Materials Science and Engineering, South China University of Technology, Guangzhou 510640, China.
2. Innovative Center for Advanced Materials (ICAM), Hangzhou Dianzi University, Hangzhou 310012, China
3. Institut des Molécules et Matériaux du Mans CNRS UMR-6283, Le Mans Université, Avenue Messiaen, Le Mans F-72085, France

Table S1. EDS results for the selected areas in the SEM images.

Samples	Position	Nd (%) (±0.1)	Ce(%) (±0.1)	Ce/RE (%) (±0.1)	Fe(%) (±0.1)	Co(%) (±0.1)	Fe/TM (%) (±0.1)	Al(%) (±0.1)
**x=0.1**	A	12.4	0.8	6.0	63.4	18.2	77.7	5.2
	B	52.4	6.9	11.6	4.2	28.5	12.8	8.0
	C	26.0	6.4	19.8	27.3	34.1	44.5	6.2
**x=0.2**	A	11.8	1.8	13.2	63.2	17.4	78.4	5.7
	B	50.5	11.5	18.5	3.0	28.0	9.6	7.0
	C	21.3	12.5	37.1	24.5	36.7	40.0	5.0
**x=0.3**	A	10.6	2.8	20.9	64.5	17.0	79.1	5.1
	B	44.7	18.6	29.4	3.0	26.8	10.1	6.9
	C	18.6	16.1	46.4	23.4	38.4	37.9	3.5
**x=0.4**	A	9.7	3.8	28.1	63.1	17.6	78.2	5.8
	B	41.6	19.6	32.0	3.8	26.2	12.7	8.8
	C	16.5	17.0	50.7	24.4	38.3	38.7	3.9
**x=0.5**	A	8.0	6.1	43.2	57.8	17.5	76.8	10.4
	B	34.7	22.7	39.5	4.8	21.3	18.4	16.4
	C	10.5	22.0	67.7	27.6	31.5	45.8	8.5
**x=0.6**	A	6.9	6.3	47.7	64.0	16.3	79.7	6.4
	B	26.4	32.4	55.1	5.7	24.2	19.1	11.5
	C	8.3	27.1	76.6	25.9	34.1	43.2	4.7
**x=0.7**	A	5.3	8.1	60.4	64.2	16.9	79.2	5.9
	B	22.9	36.6	61.5	4.0	23.7	14.4	12.9
	C	4.1	29.6	87.8	28.9	34.6	45.5	2.7

*Correspondence and requests for materials should be addressed to Z.L. (zwliu@scut.edu.cn) and L.Z. (lzzhao@hdu.edu.cn)
